# Expression proteomics study to determine metallodrug targets and optimal drug combinations

**DOI:** 10.1038/s41598-017-01643-1

**Published:** 2017-05-08

**Authors:** Ronald F. S. Lee, Alexey Chernobrovkin, Dorothea Rutishauser, Claire S. Allardyce, David Hacker, Kai Johnsson, Roman A. Zubarev, Paul J. Dyson

**Affiliations:** 1 Institute of Chemical Sciences and Engineering, Swiss Federal Institute of Technology Lausanne (EPFL), CH-1015 Lausanne, Switzerland; 2 Karolinska Institute, Division of Physiological Chemistry I, Department of Medical Biochemistry and Biophysics, Scheeles väg 2, S-171 77 Stockholm, Sweden; 3 Science for Life Laboratory, Stockholm, Sweden; 4 Protein Expression Core Facility, Swiss Federal Institute of Technology Lausanne (EPFL), CH-1015 Lausanne, Switzerland

## Abstract

The emerging technique termed functional identification of target by expression proteomics (FITExP) has been shown to identify the key protein targets of anti-cancer drugs. Here, we use this approach to elucidate the proteins involved in the mechanism of action of two ruthenium(II)-based anti-cancer compounds, RAPTA-T and RAPTA-EA in breast cancer cells, revealing significant differences in the proteins upregulated. RAPTA-T causes upregulation of multiple proteins suggesting a broad mechanism of action involving suppression of both metastasis and tumorigenicity. RAPTA-EA bearing a GST inhibiting ethacrynic acid moiety, causes upregulation of mainly oxidative stress related proteins. The approach used in this work could be applied to the prediction of effective drug combinations to test in cancer chemotherapy clinical trials.

## Introduction

Metal based anti-cancer drugs (metallodrugs) are a cornerstone of anti-cancer treatment where platinum drugs, such as cisplatin, carboplatin and oxaliplatin, are used as first line treatments for colorectal^1^, ovarian^2^, lung^3^ and bladder^4^ cancers, to name but a few. Based on their success, the development of new generations of metallodrugs has been the focus of intense research efforts encompassing various metal centres including ruthenium, rhodium, iron, iridium and gold^5^. It is well known that the use of different metal centres and ligands can tune the anti-cancer effects of metallodrugs and help to elucidate the molecular mechanism of drug activity.

**Figure 1:**
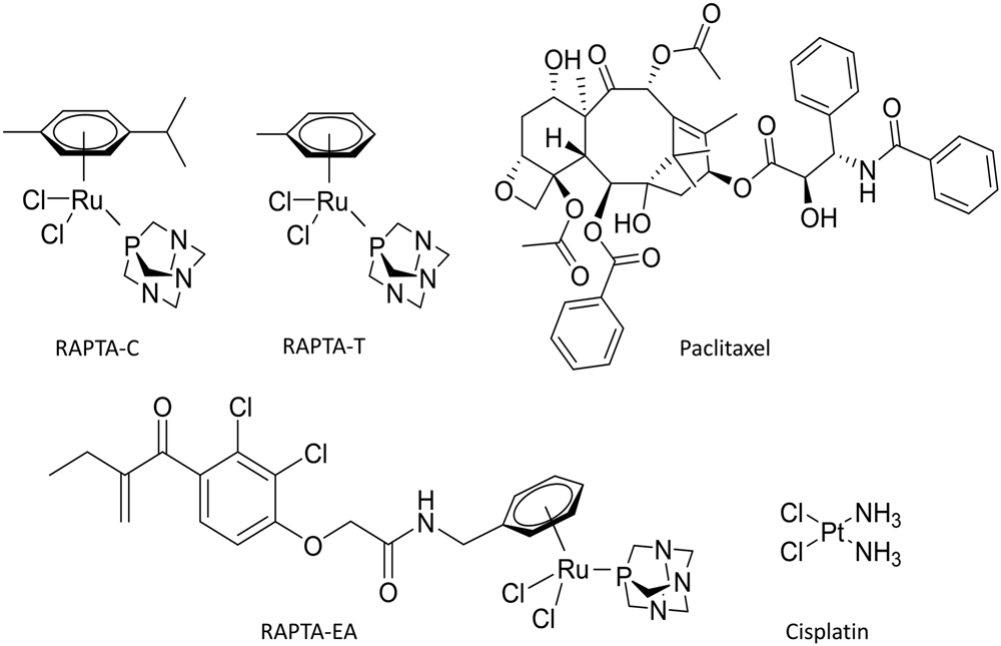
Anti-cancer compounds mentioned in this study.

Of the various alternatives to platinum-based drugs evaluated to date, ruthenium complexes have advanced furthest, with two ruthenium(III)-based compounds, namely indazolium trans-[tetrachlorobis(1H-indazole)ruthenate(III)] (KP1019) and imidazolium trans-[tetrachloro(dimethylsulfoxide)(1H-imidazole)ruthenate(III)] (NAMI-A), having been evaluated in clinical trials^6–10^. Ruthenium(III) complexes, however, are prone to ligand exchange reactions in aqueous media/physiological buffer which hamper, to some extent, the rational design of new compounds with relevant medicinal properties. Consequently, ruthenium(II)-arene compounds have attracted considerable attention following encouraging *in vivo* data on two prototypical compounds, i.e. [Ru(η^6^-*p*-cymene)Cl(en)]PF_6_, where en = ethylenediamine^11^ and [Ru(η^6^-*p*-cymene)Cl_2_(pta)], where pta = 1,3,5-triaza-7-phosphaadamantane (RAPTA-C)^12, 13^. RAPTA compounds are active on primary tumors at high doses^13^ or in combination with other drugs^12, 14^ and they also possess interesting anti-metastatic^15^ and antiangiogenic^16^ properties. The pharmacological activity of RAPTA compounds can be tuned through the introduction of various functionalities via the arene or PTA moiety^17^, with a prominent example being a RAPTA derivative in which the glutathione transferase inhibitor, ethacrynic acid (EA), is tethered to the arene ring via an amide linker^18^. The resulting compound, termed RAPTA-EA (Fig. 1), is considerably more cytotoxic than RAPTA-C, and is a more efficient GST inhibitor than EA alone and shows promising activity against certain breast cancers^19^.

Due to their very different activity profile compared to cisplatin, the biological targets of RAPTA complexes are thought to be proteins and not primarily DNA^5^. This hypothesis is supported by the observation that cellular fractionation of cancer cells exposed to RAPTA complexes show an appreciable amount of ruthenium in cytosolic and mitochondrial protein fractions^20^.

The nature of the biomolecules that metallodrugs are able to bind to is likely to be diverse^21^, however, the cellular response to these interactions is usually mediated through changes in relative protein activity. Such changes result in both the desired therapeutic effects, but also adverse events which give rise to side-effects. The change can also compensate the drugs binding interaction to restore homeostasis leading to resistance. Therefore, proteomic methods can be used to monitor the many outcomes of drug binding to chemically diverse targets. Proteomics relies on probing targets in whole cells to give a more accurate prediction of clinical usefulness. Measured protein abundances accounts not only for gene expression, but also for protein degradation and post-translational modification, and even reflects subcellular drug distribution. The dynamic range of the cellular proteome, which approaches seven orders of magnitude, may be initially limited to approximately 5000 proteins with abundance in the mass spectrometric instrumentation range^22^.

Applying filters to quantitative proteomics data allows proteome-wide drug responses to be elucidated. Filters employed include proteome subtraction, scoring, isotope enrichment, cell stage selection and fluorescent tagging, each applied singly or in combination. Proteome subtraction methods are probably most common, where the data from two experiments, e.g. two related cell lines with a different genotype or phenotype both treated with the same drug or the same cell line in the absence of a drug and incubated with a drug, are compared and, only the differences analysed. Scoring selects particular proteins based on their known cell function, ordering the significance of hits based on what is known about the role of the protein in disease progression, the desired therapeutic outcome, or the biological consequences of drug-target interactions. The scoring filter maps the proteome with respect to a specific biological response and is therefore, to some extent, like target based screening.

Proteome-wide analysis is particularly challenging to apply in metallodrug studies due to the high incidence on non-specific binding. Many metallodrugs will bind to exposed, labile amino acid residues, such as cysteine, methionine, glutamate and histidine^23^. Thus, methods based on affinity purification for protein target screening, which are extensively used for organic molecules^24^, are often inconclusive when applied to metallodrugs^25^. Similarly, using proteomics methods, even when multiple filters are applied, identifies multiple hits, some of dubious significance. These interactions are often non-specific and may also be transient, leading to poor reproducibility. Indeed, two such approaches were performed on RAPTA-type complexes^26, 27^, without clearly identifying a protein target of the drugs evaluated.

In the case of cisplatin, a number of proteomics studies have been performed, the earliest employing *E. coli* cells^28^. Subsequent studies identified a manageable number of hits from cancer cell lines, i.e. <20^29^, corresponding to proteins involved in a number of pathways, such as cellular energy metabolism, transformation, apoptosis and morphologic maintenance, and are therefore difficult to rationalise as targets versus downstream effects. Indeed, further experiments have suggested that the relatively low number of hits may have been due to detection limitations and, consequently, as technology improved, proteomics methods shortlisted hundreds of proteins modified in cells after cisplatin exposure^30^. More recently, filtering methods present a manageable number of hits that appear significant. However, in many cases the analytical success requires a prior knowledge of the drug target and the time-course evolution of the downstream effect. For example, using a combination of isotope labelling and cell cycle stage selection, proteomic analysis of cisplatin-induced apoptosis in whole cell lysates identified 26 proteins significantly upregulated of which the majority of proteins^31^ identified were known to be linked to apoptosis and of these almost half had at least one RNA-binding motif. Another study focused on drug resistance to identify protein hits consistent with expression of defence factors that protect cells from drug-induced damage^32^, including the Nrf2 mediated oxidative stress response, mitochondrial processes, protein kinases such as the targets of rapamycin (mTOR) and AMPK. In addition, specific pathways were changed by cisplatin, including eIF2 signalling of protein synthesis, actin nucleation via the ARP/WASP complex and regulation of cell polarization^33^. In each case, the data does not differentiate between direct cisplatin targets and downstream events, but indicates potential combination therapy objectives that could be used to improve the therapeutic outcome of cisplatin treatment, for example, combination therapy with rapamycin^34, 35^.

**Figure 2:**
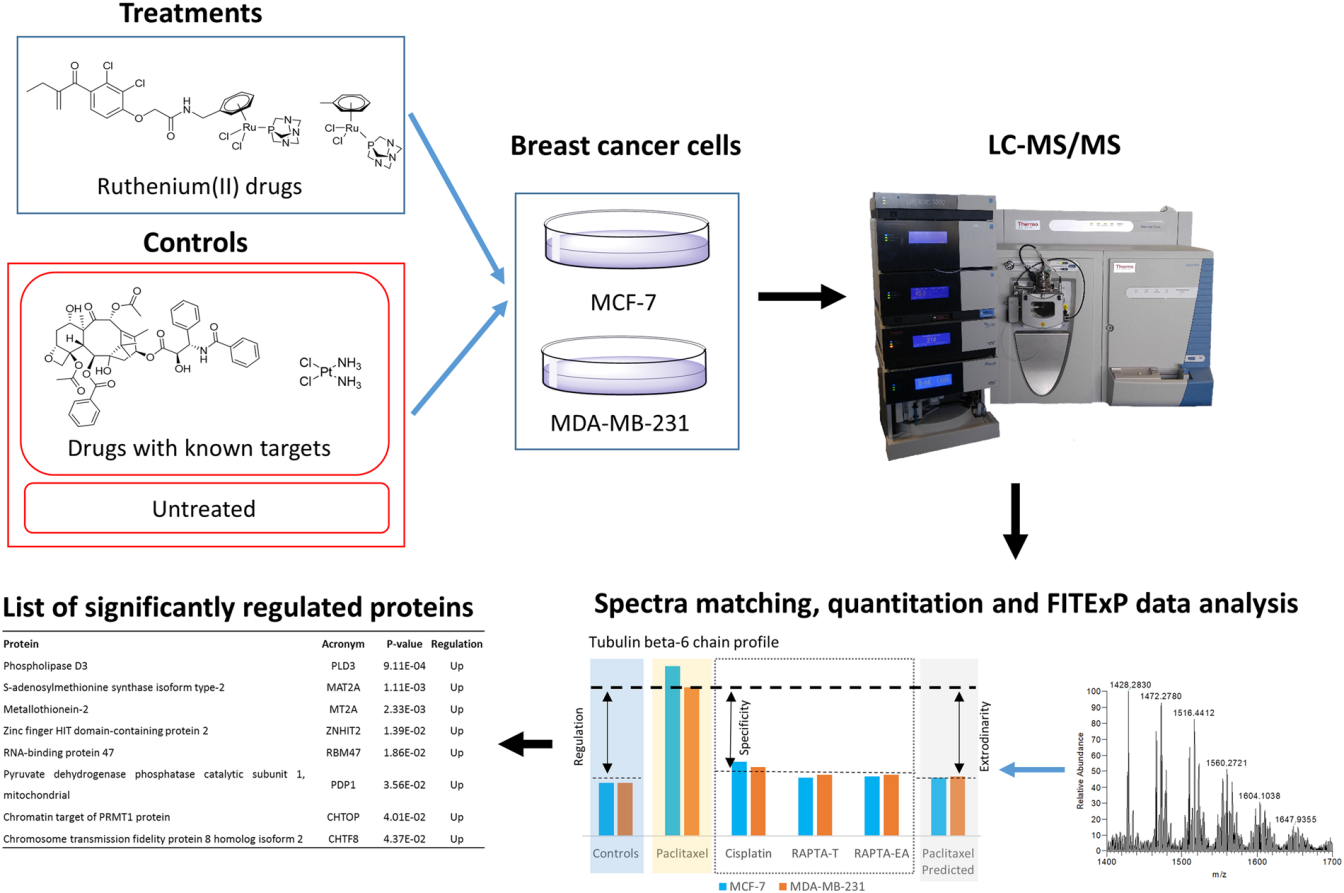
Schematic of the approach used in this study. For more details concerning the FITExP method refer to ref. 37.

Integrating quantitative pathway analysis (qPA) techniques allows the number of hits from filtered proteomics methods to be rapidly scored by relevance. With camptothecin, qPA reduced the number of hits to the known camptothecin target, TOPI, from only a handful of putative targets. Importantly, identification was possible without biasing the analysis towards known targets within the input data^36^. This method has been further advanced by introducing cell cycle stage selection, based on the observation that in late apoptosis the abundance change in protein targets of a small-molecule drug appears to be unexpectedly large compared to other co-regulated proteins^37^. The combined method, called Functional Identification of Target by Expression Proteomics (FITExP)^37^, uses protein expression data from at least two different cell lines that are referenced against positive controls, to enable the prediction of the most likely protein targets of a small molecule. This approach overcomes the limitations associated with standard proteome expression profiling methods in the identification of protein targets of anti-cancer compounds. In this work, FITExP was used to identify potential protein targets of RAPTA-T and RAPTA-EA. Two breast cancer cell lines, invasive MDA-MB-231 cells and non-invasive MCF-7 cells, were used in the analysis with paclitaxel and cisplatin as controls with known biological targets (Fig. 2).

## Results and Discussion

### Experimental validation

The FITExP method provides a list of potential drug target hits ranked in order of statistical significance with a cutoff of P < 0.05. To validate the reliability of the experimental data generated, FITExP analysis was performed with the positive controls paclitaxel and cisplatin, the former known to bind to a single protein target and the latter inducing apoptosis via DNA binding. Paclitaxel is a drug that is known to bind to tubulin promoting polymerization of microtubules leading to mitotic arrest in cells^38^. In agreement with this mechanism, FITExP analysis identified tubulin beta-6 chain as the only significantly upregulated protein.

Previous proteomic studies of paclitaxel employing selective isotope labelling^39^ identified several hundred hits that were then scored for function. One hit, Programmed Cell Death Protein 4 (PDCD4)^40^, was highlighted as the abundance of this cell-cycle regulated protein had previously been shown to be sufficient to alter paclitaxel sensitivity. The mechanism partially involves interactions with Ubiquitin-containing Enzyme E2S (UBE2S), a critical protein for mitotic exit^41^. The importance of PDCD4 was based on reports that show levels of this protein are correlated to cancer cell survival in lung cancers and the conclusion suggested the level of this protein could be used as a predictive biomarker for paclitaxel-based personalized chemotherapy^25^.

In the validation of the FITExP technique^37^ the proteomic analysis of three cell lines (melanoma A375, lung cancer H1299 and colon cancer HCT116), identified tubulins and UBE2S as significantly regulated after treatment with paclitaxel, which is in line with previous studies identifying tubulins as targets^38^ and the PDCD4-UBE2S pathway modified in response to paclitaxel. Whether the observed UBE2S up-regulation corresponds to a direct target or to a downstream effect remains to be determined, nevertheless up-regulation of this protein has been implicated in chemotherapy resistance mechanisms and, therefore, its identification suggests that combination therapies of paclitaxel and UBE2S inhibitors may be effective. UBE2S is generally highly expressed in breast cancer cell lines^42^, which may explain why it was not identified in the current study.

As mentioned above, the cytotoxic effect of cisplatin primarily involves binding to nucleophilic N-7 sites of purine bases in DNA forming interstrand and intrastrand adducts that inhibit DNA replication^43^. However, cisplatin is also known to bind to proteins^44^ and previous proteomic studies indicate that many proteins are modified by cisplatin. These proteins mainly comprise molecular chaperones^45, 46^ and proteins involved in resistance mechanisms, including detoxification^46^ and anti-apoptosis, cell structure^29, 46^ and DNA transcription^45^, that is, proteins that could be involved in response mechanism to drug toxicity rather than specific targets. FITExP analysis identified two significantly upregulated proteins, i.e. receptor tyrosine-protein kinase erbB-2 (ERBB2 or HER2/neu) and DNA damage-binding protein2 (DDB2). HER2/neu is the protein product of an oncogene which plays an important role in the development of aggressive forms of breast cancer^47^ and over expression has been linked to cisplatin resistance in various cancers in breast and lung cancers^48, 49^. Cancer chemotherapy regimens that combine the anti-HER2/neu antibody, trastuzumab, with cisplatin administration have been shown to be clinically efficacious^50, 51^. It is noteworthy that overexpression of HER2/neu is also linked to paclitaxel resistance^48^ and since this protein was not identified in cells treated with paclitaxel it may have a more important role in the overall mechanism of cisplatin. The other protein upregulated, i.e. DDB2, is involved in the nucleotide excision repair (NER) pathway^52^. DNA repair pathways have been implicated in the efficacy of cisplatin treatment, e.g. reduced levels of DNA repair proteins in testicular cancers are suggested to be linked to the effectiveness of cisplatin treatment^53, 54^ whereas upregulation of DNA repair processes are linked to drug resistance^55^. NER is a principal pathway for removing cisplatin-DNA adducts^56^ and therefore is likely to be upregulated in response to the formation of these adducts, although direct platination of DDB2 could be implicated in the mechanism of action of cisplatin.

### FITExP analysis of RAPTA-T

As mentioned above, the ruthenium(II) complex, RAPTA-T, is active against both primary and metastatic tumors at high doses and, in combination with other drugs, is effective at low doses. Moreover, the effects of RAPTA-T are more pronounced on MDA-MB-231 and MCF-7 breast cancer cells and have little impact on normal HBL-100 cells derived from breast tissue^15^.

FITExP analysis of the proteins extracted from RAPTA-T treated MDA-MB-231 and MCF-7 cells was performed using untreated cells and paclitaxel and cisplatin treated cells as controls. Table 1 shows the main proteins significantly regulated following the analysis. The top ranked protein is from the phospholipase D (PLD) family of enzymes, specifically PLD3. This family of enzymes catalyse the hydrolysis of membrane phospholipids^57^. PLD3 is poorly characterised and currently this protein is not linked to cancer progression. However, its transcript variants PLD1 and PLD2 have been shown to be involved in the progression of metastatic breast cancers^58^ and isoform-selective inhibitors of specific PLDs were shown to modulate invasiveness in metastatic breast cancer models^59^.

**Table 1:** Proteins significantly regulated following RAPTA-T treatment determined from FITExP analysis of MCF-7 and MDA-MB-231 cells.

Protein	Acronym	P-value	Regulation
Phospholipase D3	PLD3	9.11E-04	Up
S-adenosylmethionine synthase isoform type-2	MAT2A	1.11E-03	Up
Metallothionein-2	MT2A	2.33E-03	Up
Zinc finger HIT domain-containing protein 2	ZNHIT2	1.39E-02	Up
RNA-binding protein 47	RBM47	1.86E-02	Up
Pyruvate dehydrogenase phosphatase catalytic subunit 1, mitochondrial	PDP1	3.56E-02	Up
Chromatin target of PRMT1 protein	CHTOP	4.01E-02	Up
Chromosome transmission fidelity protein 8 homolog isoform 2	CHTF8	4.37E-02	Up

The second ranked protein, MAT2A, catalyses the production of S-adenosylmethionine (SAM - a key methyl donor in cellular processes) from methionine and ATP. MAT2A has been shown to be overexpressed in gastric cancers^60^ and its expression inhibition has been shown to significantly supress growth of hepatocellular carcinomas^61^. Specific inhibitors of MAT2A have also been shown to be effective agents against colorectal cancers^62^. Since plasmids for MAT2A are readily available, we expressed recombinant MAT2A to further validate RAPTA-T activity on this protein. RAPTA-T exhibited weak binding to MAT2A (IC_50_ 107 ± 13 uM) which indicated its significant upregulation is probably a downstream response to perturbations induced by RAPTA-T treatment.

Metallotheinins (MTs) are a class of proteins with high content of cysteine residues that are responsible for detoxifying heavy metals, and high levels of MT expression has been associated with poor clinical outcomes of cancer patients undergoing therapy with platinum based metallodrugs^63^. MT2 has been previously shown to bind to RAPTA-C (Fig. 1), an analogue of RAPTA-T with a closely related biological profile^64^. The binding interaction displaces the zinc ion suggesting involvement of key Cys residues. Zing finger HIT (zf-HIT) are sequence motifs found in various proteins and contain conserved cysteine and histidine residues that can coordinate zinc atoms^65^. These motifs are suggested to play important roles in gene regulation and chromatin remodelling. It was shown previously that RAPTA complexes bind proteins with zinc finger domains^25^ to displace the zinc ion^64^.

RNA binding proteins (RBMs) are proteins that bind to single or double stranded RNA and play a role in post-transcriptional control of RNAs, such as splicing, mRNA stabilization, mRNA localization and translation^66^. RBM47 has been shown to play an important role in metastatic breast cancers, where low expression of this protein is associated with highly metastatic phenotype^67^. Furthermore, RMB47 knockout mice xenografted with lung adenocarcinomas were show enhanced tumor formation and metastasis^68^. It is possible that treatment with RAPTA-T activates compensatory pathways causing increased expression of RMB47 that could be linked to anti-metastatic activity.

The pyruvate dehydrogenase (PD) complex converts pyruvate into acetyl-CoA, a substrate used in the citric acid cycle for cellular respiration^69^. Pyruvate dehydrogenase phosphatases (PDPs) in the mitochondria catalyze the dephosphorylation and reactivation of the alpha subunit of the E1 component of the PD complex^70^. PDP1 has been implicated in promoting the Warburg effect and growth in tumors such that it has been identified as a key anti-cancer target^71^. *In vitro* studies of the cellular effects of RAPTA-T have shown that the compound accumulates appreciably in the mitochondria and perturbs the expression of a large number of mitochondrial proteins, including overexpression of ATP synthetase^27^, an enzyme heavily involved in cellular respiration.

Protein arginine methyltransferases (PRMTs) catalyse the process of arginine methylation, a widespread post-translational modification in eukaryotic cells. PRMTs use S-adenosyl-L-methionine as the methyl donor, which is also a product of MAT2A. The chromatin target of PRMT1 protein (CHTOP) is a chromatin associated protein that has been shown to be critical for estrogen-dependent gene activation^72^ and is also implicated in the tumorigenicity of glioblastoma cells^73^. Considering that RAPTA-C is known to bind to histones^74, 75^, a component of chromatin, and could be a binding partner to MAT2A, it is possible that its activity also perturbs expression of CHTOP.

The chromosome transmission fidelity factor 8 (CTF8) is a chromosome cohesion protein involved in sister chromatid cohesion and fidelity of chromosome transmission^76^. It has been implicated in DNA replication and repair pathways and has been shown to have reduced expression in renal and prostate tumors^57^. Since it is a nuclear protein associated with chromatin its upregulation as a response to RAPTA-T binding to chromatin is likely, although direct RAPTA-T binding cannot be excluded.

### FITExP analysis of RAPTA-EA

RAPTA-EA is composed of the main ruthenium(II) arene fragment present in RAPTA-T, but with an ethacrynic acid (EA) moiety tethered to the arene ring (Fig. 1). EA is an inhibitor of glutathione transferases (GSTs), which are involved in the removal of exogenous substances such as cancer chemotherapeutic agents^77^, and the compound was designed to overcome GST-based resistance. Notably, GSTP1‐1 is often overexpressed in solid tumors following exposure to anti-cancer drugs^78^. RAPTA-EA shows *in vitro* GST inhibition levels superior to that of EA alone^18^ and has a much higher differential cytotoxicity than simple RAPTA-type complexes in breast cancer cell lines^19^. FITExP analysis was used to identify the main proteins regulated from RAPTA-EA treatment (Table 2). Interestingly, these hits are different from those identified with RAPTA-T treatment, suggesting the single ligand substitution dominates the biological effect.

**Table 2:** Proteins significantly regulated following RAPTA-EA treatment determined from FITExP analysis of MCF-7 and MDA-MB-231 cells.

Protein	Acronym	P-value	Regulation
Heat shock 70 kDa protein 1A/1B	HSPA 1A/1B	2.46E-05	Up
Heme oxygenase 1	HMOX1	7.44E-05	Up
TRAF-type zinc finger domain-containing protein 1	TRAFD1	5.94E-04	Up
Sulfiredoxin-1	SRXN1	2.17E-03	Up
Thioredoxin reductase 1, cytoplasmic	TXNRD1	2.21E-02	Up
DnaJ homolog subfamily B member 4	DNAJB4	3.52E-02	Up
Flavin/biliverdin reductase (NADPH)	BLVRB	4.47E-02	Up
Glucose-6-phosphate 1-dehydrogenase	G6PD	4.48E-02	Up

The top protein, heat shock 70 kDa protein 1A/1B (HSPA 1A/1B), belongs to the heat shock protein (HSP) class, which are ubiquitous and conserved proteins that act as molecular chaperones to promote correct protein folding and trafficking^70^. Some HSPs are expressed constitutively, whereas others are induced in response to specific stress^79^ and have hence been identified as potential drug targets^80, 81^. Inhibition of HSP90 has been shown to have anticancer activity; in contrast, induction of HSP70 has been linked to recovery from, for example, ischemic heart disease, diabetes and neurodegeneration^82, 83^. However, HSP70 is not thought to be an ideal drug target as reducing HSP70 activity via down-regulation can be compensated^84^. DnaJ homolog subfamily B member 4 is also a HSP that functions as both a chaperone and tumor repressor protein mainly involved in the targeting and degradation of the cell adhesion protein E-cadherin^57^. Supporting the role of HSPs in the mechanism of RAPTA-type compounds, previous non-specific proteome profiling identified HSPs as likely candidates^27^ and HSPA 1A/1B overexpression has been observed in MCF-7 cells treated with RAPTA-EA^85^.

Heme oxygenase 1 (HMOX 1) is an enzyme which cleaves heme at the alpha methane bridge forming biliverdin and is involved in hematopoesis. It is also a marker of oxidative stress, and deficiency in this protein results in impaired stress hematopoiesis resulting in marked erythrocyte fragmentation, coagulation abnormalities, and iron deposition in renal and hepatic tissues^86^. Exposure to EA has been previously shown to result in elevated expression of this protein^87^, suggesting that the observed regulation of this protein is due to the presence of the EA moiety. Flavin/biliverdin reductase was also identified and is an oxidoreductase that catalyses the NADPH-dependent reduction of biliverdin to bilirubin^70^, the next step in heme catabolism. Thus, it is not surprising to find co-regulation of this related protein in RAPTA-EA treated cells.

Sulfiredoxin-1 (SRXN1) contributes to oxidative stress resistance by reducing cysteine-sulfinic acid formed by exposure to oxidants into peroxiredoxins. As both HMOX1 and SRXN1 play a role in oxidative stress and have been shown to be co-regulated in cells exposed to anti-cancer compounds^88^, their upregulation is likely to be due to EA. Glucose-6-phosphate 1-dehydrogenase, is a cytosolic protein whose main role is the production of NADPH an electron donor in the defence against oxidising agents and in reductive biosynthetic reactions^57^. Deficiency in this protein in humans can cause neonatal jaundice and haemolysis upon exposure to oxidative stress and therefore its upregulation is likely to be linked to the cytotoxicity of RAPTA-EA. Thioredoxin reductase 1 reduces thioredoxins and other substrates and plays a role in selenium metabolism and protection against oxidative stress^57^. Depletion of GST activity is correlated to increased levels of oxidative stress response^89^, potentially leading to upregulation of this protein. TRAF-type zinc finger domain-containing protein 1 is a negative feedback regulator that controls excessive immune response in vertebrates^90^. Though not expected to be a target of RAPTA-EA, it was shown in previous studies that RAPTA-type complexes can bind to proteins containing zinc-finger domains possibly perturbing the regulation of this protein^25^.

### Implications of the FITExP analysis of RAPTA-T and RAPTA-EA

Proteins upregulated by RAPTA-EA treatment were mostly related to oxidative stress responses presumably as a result of GST inhibition by the EA moiety. Since GST levels itself were not significantly modified, and GST inhibition by RAPTA-EA has been demonstrated *in vitro* ^18^, it seems likely that these proteins are not direct targets. This would certainly seem to be the case for the proteins significantly regulated by cisplatin treatment. Cisplatin binds to DNA and regulated proteins are associated with DNA repair pathways, suggesting downstream effects in response to DNA binding. RAPTA-T shows a plethora of upregulated proteins, many of which correlate well with its toxicity to primary and metastatic tumors.

The proteins identified in these studies help to elucidate the molecular mechanism of candidate drug complexes and, importantly, should help to select other drugs that could be used in combination with them. For example, we showed that cisplatin treated cells upregulate HER2/neu which is implicated in cisplatin resistance, and cisplatin is used in the clinic in combination with trastuzumab, a HER2/neu inhibitor. Consequently, the application of RAPTA-T or RAPTA-EA in combination with known inhibitors of the key hit proteins identified in this study could be lead to effective drug combinations. From a therapeutic standpoint, RAPTA-EA could be explored in cancers where EA alone has shown potency, such as chronic lymphocytic leukemias^91^, or where EA combined with another agent shows synergy, such as the combination of EA with afatinib, an irreversible epidermal growth factor receptor tyrosine kinase inhibitors for breast cancers^92^. On the other hand, due to its broad mechanism of action, RAPTA-T could potentially be more useful if used concomitantly with drugs that target specific cancer pathways and could also play a role in therapies for later stage cancers due to its anti-metastatic properties.

### Conclusions

Here, application of the FITExP allowed us to gauge potential drug targets and mechanistic pathways involved in the action of two novel ruthenium(II) metallodrugs with very different phenotypic characteristics and from this knowledge infer their role in therapy. RAPTA-T treatment leads to upregulation of multiple proteins suggesting a broad mechanism of action involving suppression of both metastasis and tumorigenicity. In contrast, proteins upregulated upon incubation with RAPTA-EA are linked to regulation of the oxidative stress response and are therefore thought to be conferred by the EA moiety in the drug. Potentially, future application of the FITExP approach in this manner could be useful for identification of cancer chemotherapy drug combinations, where commonly cancer clinical trials fail at phase II where drug combinations are first tried and tested.

## Methods

### Materials

RAPTA-T^93^ and RAPTA-EA^18^ were prepared using literature methods. All other compounds, reagents were purchased from commercial sources and used without further purification.

### Cell maintenance, treatment and preparation for expression profiling

MDA-MB-231 (human mammary gland adenocarcinoma) and MCF-7 (human mammary gland adenocarcinoma cells) were obtained from the European Collection of Cell Cultures (Salisbury, UK). Cells were cultured in DMEM Glutamax medium supplemented with 10% fetal calf serum, penicillin 100 units/mL Streptomycin 100 µg/mL (Invitrogen). Cells were incubated at 37 °C in a moist environment containing 5% CO_2_. For proteomic expression experiments, cells were seeded at a density of 5 × 10^5^ cells in a 6-well plate for 24 h. Media was aspirated and cells were rinsed with 1X PBS before addition of fresh media containing solutions of compound (Paclitaxel, Cisplatin, RAPTA-EA and RAPTA-T, dosed at a concentration to achieve a cell kill of approximately 50% after 48 h). After 48 h, cells were detached with enzyme free cell dissociation solution and centrifuged at 200 G for 5 min. Cell pellets were snap frozen in liquid N_2_ and stored at −80 °C before sample preparation for mass spectrometry. All cell experiments were carried out in triplicate.

### Protein digestion

Breast cancer cell pellets were thawed on ice and depending on the number of cells/pellet reconstituted in 100–200 µl 8 M Urea with 100 mM NaCl. Cells were disrupted by probe sonication (Vibra-Cell™ CV18, Sonics & Materials, Newtown, USA) two times for 5/5 s cycles over 20 s followed by centrifugation at 12,000 rpm for 10 min at 4 °C. Solubilized proteins were transferred to fresh vials and the protein concentrations were determined using microBCA from Pierce (Thermo Fisher Scientific Inc). From each sample 10 µg extracted protein were dissolved in a final concentration of 0.1% ProteaseMax (Thermo Fisher Scientific Inc), 50 mM ammonium bicarbonate and 10% acetonitrile in a total volume of 80 µl. The resulting protein solutions were incubated for 45 min at 37 °C while shaking followed by an additional sonication of 10 min at room temperature. Samples were centrifuged and directly subjected to a tryptic digestion protocol carried out by a liquid handling robot (MultiProbe II, Perkin Elmer). This included protein reduction in 5 mM DTT at 56 °C and alkylation in 15 mM iodacetamide for 30 min at room temperature in the dark. Trypsin was added in an enzyme to protein ratio of 1:30 and digestion was carried out over night at 37 °C. Samples were acidified by adding 6 µl concentrated formic acid, incubated for 30 min at room temperature and centrifuged for 20 min at 3000 rpm in order to remove undigested material.

### LC-MS/MS

Tryptic peptides were cleaned with C18 StageTips (Thermo Fisher Scientific Inc) and the resulting peptide mixture was injected into a nano-Ultimate system (Thermo Scientific, Bremen, Germany) coupled to a Q Exactive mass spectrometer (Thermo Scientific, Bremen, Germany). The chromatographic separation of the peptides was achieved using a 28 cm long in-house packed column (C18-AQ ReproSil-Pur, Dr. Maisch GmbH, Germany) with the following gradient: 4–26% acetonitrile in 120 min, 26–95% ACN for 5 min and 95% ACN for 5 min all at a flow rate of 300 nl/min. The MS acquisition method was comprised of one survey full scan ranging from m/z 300 to m/z 1650 acquired with a resolution of R = 140,000 at m/z 200 and a target value of 5 × 10^6^, followed by data-dependent higher-energy collisional dissociation fragmentation scans from maximum sixteen most intense precursor ions with a charge state ≥2. Sequencing was performed with a target value of 2 × 10^5^ ions determined with predictive automatic gain control, for which the isolation of precursors was performed with a window of 4 m/z. Scans were acquired with a resolution of R = 17,500 and normalized collision energy was set to 26.

### Data processing

Fragmentation spectra were extracted using Raw2MGF (in-house developed software) and the resulting mascot generic files were searched against a SwissProt protein database (reversed protein sequences had been added to database for decoy search) using the Mascot 2.3.0 (Matrix Science Ltd.). Mascot was set up to search a concatenated SwissProt protein database (selected for Homo sapiens) with enzyme specificity set as C-terminal to arginine and lysine, allowing cleavage before proline and a maximum of two missed cleavage sites. The allowed peptide mass deviation was set to 10 ppm and 0.02 Da for the fragment ions. Carbamidomethylation of cysteine was specified as a fixed modification, whereas oxidation of methionine, N-terminal protein acetylation and deamidation of asparagine and glutamine were defined as variable modifications.

Quantitative information was extracted using in-house developed label-free software Quanti v.2.5.3.1^94^. Only reliably identified (FDR < 0.01), unmodified peptides with unique sequences were considered and only proteins discovered with at least two such peptides were quantified. For each protein, one database identifier (ID) was selected, covering all the peptide sequences identified for this specific protein. If two proteins belonging to different protein groups had a partial sequence overlap, then all the peptides belonging to this overlap were ignored. The results were reported as a set of relative protein abundances ***A*** scaled such that the geometric mean of the abundance of each protein over all samples was 1.0.

### Scoring system

For combining the data from replicate analysis, “medians of ratios” are used instead of “ratios of medians”, as has previously been suggested^95^. If relative protein abundance of *i*-th quantified protein in *c*-th cell line under *j*-th treatment is denoted as ${{\boldsymbol{A}}}_{{\boldsymbol{i}},{\boldsymbol{j}}}^{{\boldsymbol{c}}}$, then regulat*i*on ***Reg*** is calculated as:

1 $${\boldsymbol{Re}}{{\boldsymbol{g}}}_{i,j}^{c}=Median(|\mathrm{log}\,\frac{{A}_{i,j}^{c}}{{A}_{i,0}^{c}}|),$$

and specificity ***Spec*** is defined as:

2 $${\boldsymbol{Spe}}{{\boldsymbol{c}}}_{i,j}^{c}=Media{n}_{k\ne j}(|\mathrm{log}\,\frac{{A}_{i,j}^{c}}{{A}_{i,k}^{c}}|),$$

where j = 0 corresponds to untreated cells for ***Reg*** calculation, and *j≠k* for ***Spec*** calculations.

### Exceptional behaviour measure

For each *I*-th protein and each *J*-th drug treatment, two vectors were calculated:

3 $${{\boldsymbol{C}}}_{{\boldsymbol{i}}}^{{\boldsymbol{I}},\ast }=Corr\,({\boldsymbol{Re}}{{\boldsymbol{g}}}_{i,j}^{c},{\boldsymbol{Re}}{{\boldsymbol{g}}}_{I,j}^{c}),$$

4 $${{\boldsymbol{C}}}_{{\boldsymbol{i}}}^{{\boldsymbol{I}},{\boldsymbol{J}}}=Corr\,({\boldsymbol{Re}}{{\boldsymbol{g}}}_{i,j\ne J}^{c},{\boldsymbol{Re}}{{\boldsymbol{g}}}_{I,j\ne J}^{c}),$$

where ${{\boldsymbol{C}}}_{{\boldsymbol{i}}}^{{\boldsymbol{I}},{\boldsymbol{\ast }}}$ are the Pearson’s correlation coefficients of expression profiles over all treatments of *i*-th and *I*-th proteins, while ${{\boldsymbol{C}}}_{{\boldsymbol{i}}}^{{\boldsymbol{I}},{\boldsymbol{J}}}$are correlation coefficients of the expression profiles of *i*-th and *I*-th proteins excluding treatment J. Then, the linear model ${{\boldsymbol{C}}}_{{\boldsymbol{i}}}^{{\boldsymbol{I}},{\boldsymbol{\ast }}}\, \sim \,{{\boldsymbol{C}}}_{{\boldsymbol{i}}}^{{\boldsymbol{I}},{\boldsymbol{J}}}$was created and the coefficient of determination of the model was used to calculate the measure of exceptional behavior *Exc* ^*I,J*^ of *I*-th protein under *J*-th treatment:

5 $$Ex{c}^{I,J}=\,\frac{1}{{{\bar{R}}^{2}}_{I,J}}$$

### Calculation of p-values

In estimation of the p-value of a protein with a certain rank, we used the rank product method, which has previously been found to be robust and tolerant to missing values in detection differentially regulated genes in replicated experiments^96^. The method has also been successfully applied to proteomics datasets for detection of significantly regulated proteins^97^. In adaptation of the method by Schwämmle *et al*., the ***Reg, Spec*** and ***Exc*** ranks were treated as independent variables, and their values for different cell lines as well as at different incubation times were considered as independent replicate measurements. The rank product was considered to have a gamma distribution under null hypothesis, from which the p-values for the set of ranks of every protein were calculated. Adjusted p-values were calculated using standard Bonferroni correction using the total number of proteins as a multiplication factor.

### Availability of data

The mass spectrometry proteomics data have been deposited at the ProteomeXchange Consortium via the PRIDE^98^ partner repository with the dataset identifier PXD005766.

### Recombinant protein expression

Human MAT2A plasmid was obtained from Addgene (www.addgene.org, plasmid #53648) as a bacterial stab. Plasmid DNA was amplified in antibiotic-containing media and extracted with a plasmid miniprep kit (Qiagen, Basel, Switzerland) according to the manufacturer’s instructions and sequenced for validation. The plasmid was transformed into competent *E. coli* strain BL21 (DE3) Rosetta and selected with ampicillin. One colony was picked from the LB agar plate and used to inoculate a 10 mL culture of LB medium with ampicillin. The culture was grown overnight at 37 °C with agitation at 150 rpm in an incubator shaker. Next, the 10 mL culture was used to inoculate a 1 L culture of LB medium and ampicillin. The culture was incubated at 37 °C with agitation as before until an optical density of 0.6 was reached at a wavelength of 600 nm. The temperature of the incubator shaker was then reduced to 16 °C, and IPTG was added to the culture to a final concentration of 1 mM. The culture was allowed to incubate overnight at 16 °C. Next, the culture was centrifuged at 3,500 rpm for 20 min, and the cell pellets were maintained frozen at −80 °C. The frozen cell pellet was allowed to thaw and was re-suspended in 60 mL of binding buffer (150 mM NaCl and 25 mM sodium phosphate (pH 7.3)) containing a protease inhibitor cocktail (Roche Diagnostics, Rotkreuz, Switzerland). The resuspended cells were sonicated during 8 cycles of 20 s. The solution was then centrifuged at 11,000 rpm for 30 min. The supernatant was retained and imidazole was added to 10 mM. Then 2 ml of FastFlow IMAC beads (GE Healthcare, Glattbrugg, Switzerland) were added. The solution was mixed by rotation for 1 h at 4 °C. The resin was transferred to a disposable column (Bio-Rad, Crissier, Switzerland) and washed with 10 column volumes (CVs) of binding buffer with 10 mM imidazole. Next, the column was washed sequentially with 10 CVs of binding buffer with 25 mM imidazole, 5 CVs of binding buffer with 50 mM imidazole, and 5 CVs of binding buffer with 100 mM imidazole. The protein was then eluted with 4 × 2 CVs of binding buffer with 250 mM imidazole. The washes and elutions were analyzed by reducing SDS-PAGE. The fractions with the recombinant protein were pooled and dialyzed twice against 2 L of PBS. After dialysis the concentration of the protein was determined by absorbance at 280 nm.

### MAT2A enzymatic assays

Recombinant human MAT2A stored in 50% glycerol was dialysed into MAT2A buffer containing 50 mM MOPS at pH 7.4, 50 mM potassium acetate, 20 mM magnesium acetate. All substrates and compounds used were dissolved in MAT2A buffer. MAT2A was pre-incubated with different concentrations of RAPTA-T (1000, 500, 250, 125, 62.5, 31.3, 15.6, 7.8 and 0 µM) at various time-points at 37 °C. 50 µM L-methionine and 1 mM ATP were added to start the reaction. Final reaction volumes were fixed at 150 µL. After 30 min, reactions were quenched with 5 µL acetic acid and cooled on an ice pellet. 25 µL of sample was used for a phosphate colorimetric assay (Sigma-Aldrich, MAK030) according to the manufacturer’s instructions, and the IC_50_ values were calculated in Graphpad Prism version 6.00 for Windows. The IC_50_ values reported were the mean of two independent experiments.
